# Data Model for the Comprehensive Management of Biobanks and Its Contribution to Personalized Medicine

**DOI:** 10.3390/jpm14070668

**Published:** 2024-06-21

**Authors:** Ana María Sánchez-López, Purificación Catalina, Fernando Franco, Sonia Panadero-Fajardo, Juan David Rejón, María Concepción Romero-Sánchez, Jose Manuel Puerta-Puerta, Rocío Aguilar-Quesada

**Affiliations:** 1 Andalusian Public Health System Biobank, Coordinating Node, 18016 Granada, Spain; purificacion.catalina@juntadeandalucia.es (P.C.); fernando.franco@juntadeandalucia.es (F.F.); sonia.panadero@juntadeandalucia.es (S.P.-F.); juand.rejon@juntadeandalucia.es (J.D.R.); mconcepcion.romero.sanchez@juntadeandalucia.es (M.C.R.-S.); josem.puerta.sspa@juntadeandalucia.es (J.M.P.-P.); rocio.aguilar.quesada@juntadeandalucia.es (R.A.-Q.); 2 Instituto de Investigación Biosanitaria Ibs.GRANADA, 18012 Granada, Spain; 3 Unidad de Gestión Clínica Hematología y Hemoterapia, Hospital Universitario Virgen de las Nieves, 18014 Granada, Spain

**Keywords:** biobank, personalized medicine, data model, data harmonization, BIMS

## Abstract

Biobanks are infrastructures essential for research involving multi-disciplinary teams and an increasing number of stakeholders. In the field of personalized medicine, biobanks play a key role through the provision of well-characterized and annotated samples protecting at the same time the right of donors. The Andalusian Public Health System Biobank (SSPA Biobank) has implemented a global information management system made up of different modules that allow for the recording, traceability and monitoring of all the information associated with the biobank operations. The data model, designed in a standardized and normalized way according to international initiatives on data harmonization, integrates the information necessary to guarantee the quality of results from research, benefiting researchers, clinicians and donors.

## 1. Introduction

A widely accepted definition of biobank includes the management of samples (biological materials) but also their related information and data [1,2], playing a crucial role in biomedical research by providing biological samples and associated data for scientific purposes. Biobanks are professionalized infrastructures that require increasingly complex governance models involving many stakeholders, including donors, researchers, clinicians, funders and industry, playing a central role in sustainability [3]. In addition, the governance of biobanks must be adapted to the needs of and trends in research, with the highlighted role of next-generation biobanking for personalized medicine [2]. Indeed, key areas of expansion of precision medicine have been identified [4], of which biobanks will be a part.

In this context, one of the cutting-edge factors impacting biobank activity is digitalization and virtualization, with biobanks of images for precision medicine being a clear example [5]. Virtualization has innumerable advantages for biobanks, with digitized information associated with or derived from the analysis of samples from different platforms for biomedical research. It adds value to the samples available upon becoming infinite and perpetual resources and that are still available even after the sample is finished. Digitalization and virtualization allow for better and more sustainable preservation of the sample that becomes an archived digital image; it does not deteriorate and it does not lose integrity due to the storage. However, virtualization makes it necessary to have a biobank information management system (BIMS), a robust tool used to record, trace and monitor all the information associated with the samples and biobank operations, some of whose functioning has been previously reported [6].

The diversity in nature and the format of collected data involves significant challenges for the biobanks. Through the effective implementation of standards in the data acquisition, coding and management process, the quality and thereby confidence in data integrity is improved. Furthermore, standardization enables interoperability between different systems, facilitating international collaboration by integrating data and comparing them among biobanks from different countries and regions. The following are the most commonly used general and specifically developed standards in biobanking data coding and harmonization:

- ICD (International Classification of Diseases) [7]: A classification system used to classify and code diseases, disorders, injuries and causes of death, as well as other health conditions of donors.
- SNOMED-CT (Systematized Nomenclature of Medicine-Clinical Terms) [8,9]: A medical coding system based on a hierarchical system of concepts and semantic relationships used to encode data on types of biological samples, diseases, laboratory procedures, medical treatments and other relevant clinical entities.
- OMOP (Observational Medical Outcomes Partnership) [10,11,12,13]: A data model designed to standardize and analyse clinical data in observational studies, providing a standard framework for data structure and nomenclature. It allows us to store medical data with standardized terminologies. Furthermore, it includes several applications that enable the analysis of patient data for different research purposes.
- BRISQ (Biospecimen Reporting for Improved Study Quality) [14]: A set of recommendations structured into three levels that provide detailed guidelines on how to document and present information about biological samples in biobanks.
- MIABIS (Minimum Information About Biobank Data Sharing) [15,16,17]: A set of standards defining the minimum information required to describe a biobank and its samples.
- SPREC (Sample PREanalytical Code) [18,19,20,21]: A seven-element code designed for providing details about preanalytical variables including the collection, processing and storage of fluid and solid biological samples.

Additionally, the application of FAIR principles (Findable, Accessible, Interoperable, Reusable) during the standardization process is widely recommended by international organizations and institutions to facilitate the use of data by the scientific community in research projects and promote interoperability [22].

Considering all the above, the SSPA Biobank has implemented a comprehensive BIMS made up of different modules that allow for the recording, traceability and monitoring of all the information associated with the biobank operations, including samples, donors and requests. The BIMS data model, described in this work, has been designed in a standardized and normalized way according to previous international initiatives on data coding, harmonization and access and considering the recommendations in data collection and their management provided by different initiatives and organizations [23,24]. Questionnaires and additional references were also used when necessary for the establishment of specific datasets [25,26].

## 2. Design of the Data Model and Their Integration in the Information Management System: Relationship of the Information and Exploitation

The SSPA Biobank’s BIMS, called nSIBAI, has been co-developed by the Andalusian Public Health System and the company Biosoft Innovation S.L. The technology used for its development, the Mongo DB data system, gives it great capacity and versatility to incorporate new functionalities and adapt the data model through a relatively simple configuration while maintaining the necessary security. In addition, it allows integrations with other software to facilitate access to additional information and speed up all the tasks performed [27]. Currently, nSIBAI is working in more than 20 nodes of the SSPA Biobank, operating as a virtual biobank [28,29].

nSIBAI is organized in different modules that are connected between them. The principal modules which integrate the BIMS are as follows:

- Donor management: donors with sample donations to the Biobank, and potential donors registered in the Andalusian Registry of Donors for Biomedical Research (REDMI) [27].
- Sample/donation management: this includes three main areas: (1) collection of samples and data (donations), (2) sample processing data and (3) stored sample data.
- Request/project management: this module is organized in four areas:
  - Legal, ethical and administrative management data related to the requests.
  - Follow-up of sample and data acquisition.
  - Deliveries of samples and data to the researchers/users.
  - Return of research results.

**Figure 1:**
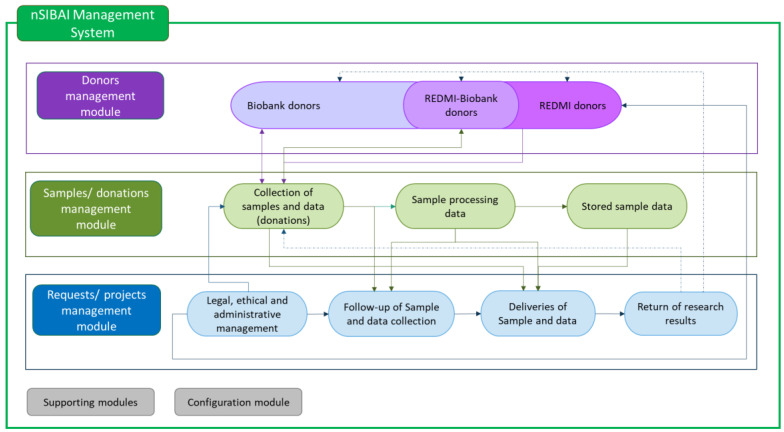
Interaction of main modules of nSIBAI.

The interrelationship of these modules allows the SSPA Biobank to manage information in order to integrate all the activities and offer a quality service for research. Moreover, the BIMS has supporting and configuration modules (Figure 1).

**Figure 2:**
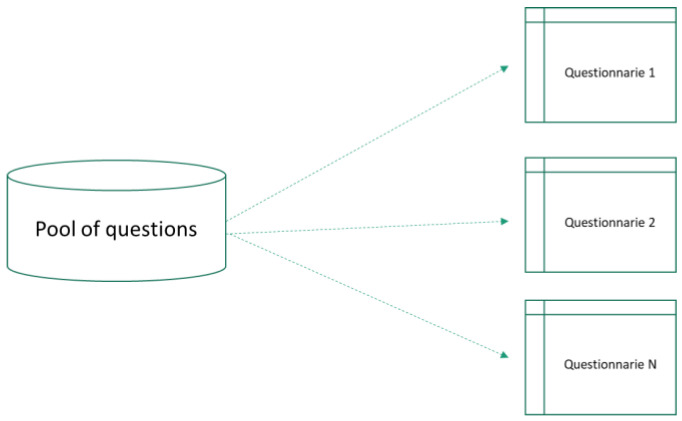
N questionnaires of nSIBAI are created from a pool of questions in response to the information to record.

To record the information of each module, the BIMS has fields directly associated with the items or questionnaires. Questionnaires are organized in at least four groups: (1) donors, (2) donations, (3) samples and (4) processes. All of them are designed by specific staff from the SSPA Biobank from a pool of questions, and they are capable of creating new questions and questionnaires and modifying them in order to adapt them to new needs of the Biobank related to biomedical research (Figure 2). To guarantee that critical data are available and consistent, a procedure for the quality control of data has been developed.

Relevant fields and questions corresponding to the modules of nSIBAI’s data model are shown as tables, classified by but not strictly related to the following:

- Donor-associated data (Table 1).
- Donation-associated data (Table 2).
- Data related to the protection of the rights of donors (Table 3).
- Sample and process-associated data (Table 4).

The tables show the information collected (data group and data description), the type of data (text, number, list or date), the way of recording data (field, questions included in a questionnaire or automatically filled in by the BIMS) and if these data or related information are part of BRISQ, MIABIS, OMOP or SPREC standards as references for data harmonization (√). On the other hand, when this information is used to access samples through the catalogue of the SSPA Biobank, it is indicated.

**Table 1:** Donor-associated data.

Data Group	Data Description	Type of Data	Recording	BRISQ	MIABIS	OMOP	Catalogue
Donor identification	Sample donor ID	Numeric	Automatic code		√	√	
	Type and Number ID	List, Number	Fields				
	First and Last Name	Text	Fields				
	Clinical number ID	Number	Fields				
Demographic data	Sex	List	Field	√	√		√
	Age	Number	Field	√	√		√
	Birth date	Date	Field		√	√	
	Race	List	Field			√	
	Ethnicity	List	Field			√	√
	Country of birth	List	Field				
	City and state of birth	List	Field				
	City and state of residence (*n* times)	List	Field				
Contact information	E-mail	Text	Field				
	Landline or Mobile Phone	Number	Fields				
	Address	Text	Field				
	ZIP	Number	Field				
	State	List	Field				
	City	List	Field				
	Country	List	Field				
Health data (information collected through donor)	Disease status	Text	Field	√			
	Clinical or pathology diagnosis	Text	Field	√		√	
	Diagnosis date (chronic diseases)	Text	Field	√		√	
	Clinical characteristics	Text	Field	√			
	Epidemiological characteristics	Text	Field				
	Family’s medical history	Text	Field				
Kinship relations	Family ties	List	Field				
Recruitment information	Name of divulgation event	List	Field				
	Member of patient associations	List	Field				

**Table 2:** Donation-associated data.

Data Group	Data Description	Type of Data	Recording	BRISQ	MIABIS	OMOP	Catalogue
Donation identification	Donation ID	Alphanumeric	Automatic code		√		√
	Collection (event) date and time	Date/Time	Field		√		
	Source code	Text	Field				
	Visit concept	List	Field			√	√
	Visit concept value	Number or list	Field			√	√
	Age at event	Auto calculated	Automatic	√	√		√
	Care source unit	List	Field			√	
Clinical data: pathological or control	Diagnosis CIE-10	List	Questionnaire	√	√		√
	Health control group	List	Questionnaire	√	√		√
	Diagnosis SNOMED-CT	List	Questionnaire	√	√		√
	Diagnosis SNOMED II	Text	Questionnaire	√	√		√
	Non-codified diagnosis	Text	Questionnaire	√	√	√	
	Disease status	List	Questionnaire	√	√	√	
	Debut date	Date	Questionnaire		√		
	Diagnosis date	Date	Questionnaire	√	√	√	
Clinical data: treatment and follow-up	Treatment	List	Questionnaire	√	√	√	
	Treatment type	List	Questionnaire	√	√	√	
	Treatment date	Date	Questionnaire		√	√	
	Treatment response	Text	Questionnaire		√	√	
	Disease-free survivability	Number	Questionnaire				
	ECOG scale	List	Questionnaire				
Health perception	General health perception	Text	Questionnaire		√		
	Health perception compared to others	Text	Questionnaire				
	Health perception compared to last year	Text	Questionnaire				
	Health-related limitations	Text	Questionnaire				
Lifestyle and consumption habits	Dietary habits	Text	Questionnaire		√		
	Exercise frequency	Text	Questionnaire		√		
	Regularity of alcohol-drinking	Text	Questionnaire		√		
	Tobacco consumption	List	Questionnaire		√		
	Another drugs consumption	List	Questionnaire		√		

**Table 3:** Data related to the protection of the rights of donors.

Data Group	Data Description	Type of Data	Recording	MIABIS
Informed consent	Signed consent date	Date	Field	
	Consent file	Archive	Field	
	Legal representative ID	Number	Field	
	Legal representative information (name and surname)	Text	Field	
	Professional ID involved in the information process	Number	Field	
	Professional identification (name and surname)	Text	Field	
	Collection method	List	Field	
	Detail of collection method	Text	Field	
	Identification of sample (codified or anonymized)	List	Field	
	Consent to contact later	List	Field	
	Ways to contact	List	Field	
	Detail of contact (phone, email…)	Text	Field	
	Consent to receive genetic or other health relevant information	List	Field	
	Authorized research areas, education or quality control	List	Field	
	Use restrictions	Text	Field	√
Revocation	Revocation date	Date	Field	
	Revocation type (partial or total)	List	Field	
	Revocation file	Archive	Field	
	Other considerations	Text	Field	

**Table 4:** Sample-associated data.

Data Group	Data Description	Type of Data	Recording	BRISQ	MIABIS	OMOP	SPREC	Catalogue
Sample identification	Sample ID	Alphanumeric	Automatic code		√	√		√
	Source code	Text	Field					
Applied process data	Process applied ID	Alphanumeric	Automatic code					
	Process applied name	List	Field	√				
	Start date and time	Date/time	Field	√			√	
	End date and time	Date/time	Field	√			√	
Pre-analytical data	Type of sample	List	Field	√	√	√	√	√
	Sample characteristics	List	Field					√
	Type of cellular line	List	Field	√	√			√
	Anatomical site	List	Field	√	√	√		√
	Quantity of sample (volume or size)	Number	Field	√		√		√
	Container	List	Field	√			√	√
	Additive	List	Field	√			√	√
	Collection date and time	Date/time	Field			√		
	Type of collection/collection mechanism	List	Field	√			√	
	Reception temperature	Number	Field	√				
	Warm ischemia time	List	Questionnaire	√			√	
	Cold ischemia time	List	Questionnaire	√			√	
	Cold ischemia temperature	List	Questionnaire	√			√	
	Fixation time	Number	Questionnaire	√			√	
	Reception date and time	Date/Time	Field	√				
	Pre-centrifugation delay	List	Questionnaire	√			√	
	Pre-centrifugation temperature	List	Questionnaire	√			√	
	Centrifugation speed	Number	Questionnaire	√			√	
	Centrifugation time	Number	Questionnaire	√			√	
	Centrifugation temperature	Number	Questionnaire	√			√	
	Centrifugation: stroke	List	Questionnaire	√			√	
	Post-centrifugation delay	List	Number				√	
	Post-centrifugation temperature	List	Questionnaire				√	
	Freezing method	List	Questionnaire	√				
	Freezing temperature	List	Questionnaire	√				
	Long-term storage temperature	List	Questionnaire	√	√		√	
	Long-term storage container	List	Questionnaire	√			√	
	Start date and time of storage	Date/Time	Field	√				
Quality/Analytical data	Thawing method	List	Questionnaire	√				
	Cellular viability (%) and others related	Number	Questionnaire	√				
	Medium of culture	List	Questionnaire	√				
	Method of acid nucleic extraction	List	Questionnaire	√				
	Method of quantification	List	Questionnaire	√				
	Ct value (specific for each PCR: flu, SARS-CoV, VPH…)	Number	Questionnaire	√	√	√		
	Concentration and others related	Number	Questionnaire	√				
	RIN	Number	Questionnaire	√	√			
	DIN	Number	Questionnaire	√	√			
	Immunohistochemical study (Ab and result)	List	Questionnaires	√	√			
	Histochemical study (staining and result)	List	Questionnaires	√				
	Value of STRs	Number	Questionnaire					
	Chromosome and genetic identification method	List	Questionnaires	√	√			
	Chromosome formula and others related	Text/number	Questionnaires	√	√			
	Image of karyotype	Archive	Questionnaire	√	√			
	Biochemistry parameters (cholesterol, LDL, Protein C, Vitamin D, Glucose…)	Number	Questionnaires	√		√		
	Screening (positive/negative) microbiological agents (Herpes, SARS-CoV-2, Citomegalovirus, …)	List	Questionnaires	√		√		
	Haematological parameters (lymphocytes, erythrocytes, …)	Number	Questionnaires	√		√		
	Histological evaluation	List/Text	Questionnaires	√				
	Histological grade	List	Questionnaire	√				
	Technical report	Archive	Questionnaire	√				

## 3. Provision of Samples and Associated Information and Return of Research Results

Researchers can access SSPA Biobank’s collections of samples and associated data through a well-defined procedure. Briefly, the requests are made through a form in which researchers describe the needs of samples and/or associated data for a project previously approved by an ethical committee. The Biobank evaluates whether the samples are already available in the stock of the SSPA Biobank and whether the associated data are recorded, or if a prospective collection of samples or an update of data are also necessary. Prior to providing the samples and data required by the project, the Biobank will require approval from its external ethics and scientific committees, which includes the number and type of samples and associated data being fit for purpose, in compliance with specific Spanish national laws (Law 14/2007 on Biomedical research and Royal Decree 1716/2011 on Biobanks and Human Biological Samples). Both committees provide an additional guarantee of equitable access to the biospecimens and data by the researchers according to the rights of donors. Finally, a Material Transfer Agreement (MTA) is signed between the SSPA Biobank and the researcher before sending samples. Data associated with samples are provided to researchers by email as a confidential file attached in compliance with EU General Data Protection Regulation 2016/679 and the specific Spanish national Organic Law 3/2018 on the Protection of Personal Data and Guarantee of Digital Rights. This process records and monitors using the SSPA Biobank BIMS, and it will be possible to request the biospecimens using this tool in the near future.

MTA includes, among other things, the obligation of the researcher to inform the Biobank of the genetic results relevant for the health of donors and incidental findings, as well as any scientific publication or technical document, communication and intellectual or industrial property document performed with the samples and associated data provided. The information related to research results is recorded through a dataset composed of different fields, some of them general for all research results and others specific for each type (Table 5). The importance of the recognition in communications of the biobanks as support infrastructures has been highlighted by the community, and a specific initiative, the Bioresource Research Impact Factor/Framework (BRIF), was even developed to quantitatively evaluate the impact of the use of bioresources in research [30,31]. Consequently, CoBRA (Citation of BioResources in journal Articles) was considered to identify the research results mentioning the SSPA Biobank to record in the BIMS associated with the corresponding provision [32,33].

As an added value, the interaction between the biobank and the researchers can be based on a win-win relationship. MTA could be extended based on a specific governance model associating the raw data (e.g., genomics, metabolomics, and images) from analysis developed by the researcher to the samples and contributing to the virtual biobank. These types of data could be included in the BIMS through different strategies: storing the data in the BIMS or linking with the original source of data for access through specific identifiers.

## 4. Conclusions

The BIMS data model of the SSPA Biobank has been designed and adapted in compliance with the available standards, integrating the information necessary to guarantee the Biobank operations. The benefits for researchers are related to the reproducibility of research thanks to the better annotation of specimens and the availability of an increasing amount of associated data useful for research as well as the access to samples through the virtual catalogue. Clinicians collecting samples will also have all this information available, including incidental findings, making it possible to achieve better diagnostics and treatments of their patients. The benefits for donors derive from advances in health and from an exhaustive characterization of their samples allowing for precision medicine. However, in the frame of the Quality Management System of the SSPA Biobank, the BIMS data model will continue to improve by adding or supporting new attributes reported by the international community.

## Figures and Tables

**Table 5:** Data model for publications or technical documents, communications and intellectual or industrial property documents as research results performed with samples and associated information provided by the Biobank.

Data Group	Data Description	Type of Data
Common data	Summary of project results (divulgation version)	Text
	Summary of project results	Archive
	Classification of research result (congress, publications or industrial/intellectual property)	List
	Title of result	Text
Publications or technical scientific documents (articles, book, guides, thesis…)	Authors	Text
	Mention of Biobank	List
	Publication type	List
	Corresponding author	Text
	Name of journal	List
	Indexing (Impact Factor, Quartil, Decil)	Number
	Publication year	Number
	Publication date	Date
	Publication pagination (volume/number/pages)	Number
	Publication registration ID (PMID/ISSN/DOI/ISBN)	Number
	Link to publication	Special Text
	Research result	Archive
Communications	Authors	Text
	Congress/conference/symposium name	Text
	Magazine publication	List
	Place of celebration (city and country)	Text
	Date of celebration	Date
	Organizer entity	List
	Even type	List
	Type of participation	List
	Geographical scope	List
	Research result	Archive
Industrial and intellectual property	Type of industrial property	List
	Owner/s	Text
	Inventor/s	Text
	Rights holder entity	Text
	Application date	Date
	Application number	Text
	Country of registration	Text
	Registration date	Date
	License concession date	Date
	Protection mode	List
	Patent ID	Text
	PCT Patent	Text
	Spanish patent	Number
	Country of property	List
	Exploitation status	List

## Data Availability

The data produced in this study are shown in this article.
